# Gastric cancer and related epigenetic alterations

**DOI:** 10.3332/ecancer.2017.714

**Published:** 2017-01-17

**Authors:** Trupti N Patel, Soumyadipta Roy, Revathi Ravi

**Affiliations:** Department of Medical Biotechnology, VIT University, Vellore 632014, Tamil Nadu, India

**Keywords:** gastric cancer, epigenetics, histone modification, methylation, microsatellite instability

## Abstract

Gastric cancer, a malignant and highly proliferative condition, has significantly affected a large population around the globe and is known to be caused by various factors including genetic, epigenetic, and environmental influences. Though the global trend of these cancers is declining, an increase in its frequency is still a threat because of changing lifestyles and dietary habits. However, genetic and epigenetic alterations related to gastric cancers also have an equivalent contribution towards carcinogenic development. DNA methylation is one of the major forms of epigenetic modification which plays a significant role in gastric carcinogenesis. Methylation leads to inactivation of some of the most important genes like DNA repair genes, cell cycle regulators, apoptotic genes, transcriptional regulators, and signalling pathway regulators; which subsequently cause uncontrolled proliferation of cells. Mutations in these genes can be used as suitable prognostic markers for early diagnosis of the disease, since late diagnosis of gastric cancers has a huge negative impact on overall patient survival. In this review, we focus on the important epigenetic mutations that contribute to the development of gastric cancer and the molecular pathogenesis underlying each of them. Methylation, acetylation, and histone modifications play an integral role in the onset of genomic instability, one of the many contributory factors to gastric cancer. This article also covers the constraints of incomplete knowledge of epigenetic factors influencing gastric cancer, thus throwing light on our understanding of the disease.

## Introduction

Gastrointestinal malignancies rank as the second most death causing condition, although with lower incidences than certain other cancers globally [1–3]. Gastric cancer begins when the cells in the inner lining of the gut become cancerous and lead to aberrant proliferation resulting in ulceration, inflammation, and ultimately leading to tumour formation. Usually it grows slowly over the years and is generally undetectable in the early stages, delaying diagnosis of the disease. Although with the recent advances in diagnostics and therapeutics, the mortality rate of gastric cancer has declined significantly, but it still remains difficult to cure as most patients get diagnosed at a very advanced stage. The patients who are diagnosed at an early stage and subsequently undergo therapy or surgeries often have relapses, and the unavailability of advanced treatments ultimately leads to mortality [4]. The factors influencing the onset and progression of the disease range from dietary habits and environmental influences and infections to underlying epigenetics factors and certain diagonalisable genetic alterations. In this review, we touch on all the factors with special emphasis on epigenetic influence as the contributing factor towards gastric cancers.

## Aetiology—from unknown to known

Gastric carcinoma is a multifactorial disease with environmental and dietary factors being the pivotal contributors in the incidence of the disease [5]. The most significant risk factors are listed below:

### Helicobacter pylori (H. pylori) infection

In 1994, The International Agency for Research on Cancer identified *H. pylori* as a type 1 carcinogen in human beings [6]. *H. pylori* is a gram negative bacterium growing on the stomach mucosa in chronic gastritis. They are not generally seen in normal stomach lining but colonise in the stomach in cases of chronic bacterial infections [7–8]. *H. pylori* infections are most likely to be acquired during childhood through oral ingestion [9] and can cause an inflammatory reaction in the host’s immune response which is found to disappear on treatment with antibiotics thereby strongly supporting it as an important environmental agent in the occurrence of gastric cancer [7, 10]. If untreated, prolonged inflammations lead to the production of toxins and also cause oxidative stress followed by rapid cell proliferation, progressive development of gastric lesions from chronic gastritis, finally resulting in gastric carcinogenesis [9]. Inflammation associated with carcinogenesis caused by *H. pylori* has other mechanisms too. A previous study by Piazuelo *et al* [8] revealed that oxidative DNA damage caused by certain reactive oxygen species leads to hypermethylation of CpG islands in the promoter region of some tumour suppressor genes like reprimo (RPRM) thereby silencing them and leading to cancer initiation.

### Dietary risks

High salt content in diet and smoked or poorly preserved food items are associated with the development of gastric carcinomas [8]. Earlier, studies revealed that prolonged consumption of salty or pickled food and/or sundried food causes atrophic gastritis which leads to alteration in the gastric mucosa by formation of N-nitroso compounds [11]. It also promotes the growth of nitrosating bacteria and catalyses the production of the carcinogenic N-nitroso compounds which form DNA adducts and if not removed can lead to G:C→A:T transversions [9], thereby altering the DNA sequence and causing genomic instability over a period of time. Lately, metagenomics data has proved that changes in the microbiota of the gut has a prolonged influence on the various gastric related conditions and finally contribute towards tumourigenesis.

### Lifestyle

Following an erratic lifestyle for a prolonged period of life is an important causative factor of gastric cancer. Studies have revealed that approximately 18% of stomach cancer cases can be traced to tobacco smoking. Cigarette smoking increases the risk of proximal gastric cancer by 2–3 times [11]. Little or no correlation has been found between alcohol consumption and gastric cancer, however, subjects with chronic alcohol consumption are found to have increased risk in gastric cancer related deaths [11]. Obesity is also a potent risk in the incidence of gastric cancer. A recent study in USA revealed that the risk of death because of gastric cancer can be correlated with high body mass index in males [9]. Apart from this, genetic susceptibility also accounts as a major contributing factor in gastric cancer development.

### Inheritance

Literature studies have revealed that the Adenomatous Polyposis Coli [APC] gene responsible for causing Familial Adenomatous Polyposis (FAP) is mutated at a high frequency in gastric cancer. The risk of gastric cancer in FAP patients is ten folds as compared to the general population. Moreover, well differentiated types of gastric cancers are found to originate from the intestinal metaplastic regions of the gastric mucosa and frequent loss of heterozygosity (LOH) can be seen on Chromosome 5, with APC gene located in the *q* arm of the chromosome [12].

Hereditary Diffuse Gastric Cancer (HDGC), an inherited condition caused because of mutation in the E-cadherin gene CDH1 is associated with increased risk of gastric cancer. It is an autosomal-dominant syndrome in which the affected individuals develop diffuse type gastric cancer at an early age. However, the emergence of gene-directed gastrectomy allowing for curative surgery at an early stage and advanced diagnostic measures have reduced the cancer related deaths caused because of HDGC [13].

### Other factors

As reported, gastric cancer is a disease with various causative agents. Apart from the abovementioned causes, there are also a few less commonly known factors that may contribute to its development. According to the OMIM database, 10% of gastric cancer cases are hereditary and a family history of genetic syndromes like Lynch syndrome or HNPCC (hereditary nonpolyposis colorectal cancer) and Li-Fraumeni syndromes related to APC and p53 respectively cause a significant increase in cancer related risk [14]. Some other factors include mutagenic radiations, pernicious anemia [8], Epstein-Barr virus [9], and importantly emerging epigenetic changes like DNA methylation, histone modifications, and miRNA deregulation.

### Role of epigenetics in cancer

The concept of epigenetics was first put forth by C H Waddington who defined it as 'the causal interactions between genes and their products, which bring the phenotype into being' [15, 16]. Epigenetics, today is defined as the study of heritable changes in gene expression which are not influenced by any modification in the primary DNA sequence [16]. A few important epigenetic processes associated with most of the cancers are; DNA methylation, histone modification, and modification of non-coding RNAs including miRNAs [16]. Together, these processes can lead to underexpression or overexpression of genes. This leads us to explain the term epimutation which is defined as the process which causes abnormal suppression of active genes or abnormal triggering of inactive genes [17].

In humans, DNA methylation occurs in CpG rich sites called CpG islands which are found to be present at the 5’ end of many regulatory genes [15–18]. In normal cells, DNA methylation significantly modulates the gene expression by female X-chromosome inactivation and genomic imprinting, whereas in cancer cells it is altered to a great extent because of loss of imprinting. Depending on the number of methyl groups, methylation can cause excessive activation of genes by hypomethylation or inactivation of genes by hypermethylation, both of which lead to cancer by either activation of oncogenes or repression of tumour suppressor genes.

Histone modification, which also triggers the oncogenes or suppresses the tumour suppressor genes, is dependent on the residue type which is modified and the form of modification present. Acetylation, for instance, causes transcriptional activation while methylation may cause transcriptional activation or repression based on the type and location of residue altered and also the extent to which methylation has taken place [15, 16].

While most studies are focused on the genes coding for proteins, it has recently been found that the part of the genome not coding for any proteins, i.e. the non-coding RNA which includes small non-coding RNAs (like microRNA or miRNA) and long non-coding RNAs 9 (lncRNA), also plays a considerable role in the occurrence of the disease [19]. The aberrant expression of miRNA is linked with anomalous epigenetic regulation and is explained later in the article.

## Epidemiology—Indian and global scenario

The risk of gastric cancer increases with age with peak incidence between 55–80 years, while cases of gastric adenocarcinoma are rare in patients below 30 years [8, 14]. A study by Saha *et al* showed the male to female ratio to be 2.7:1 in gastric cancer patients of West Bengal State in India, with male predominance in patients above 40 years [20]. Another study revealed that in the Indian subcontinent the incidence of gastric cancer is found to be higher in certain economically deprived communities than the others. This is probably because irregular dietary habits and increased alcohol and tobacco consumption of low grade in this population. In India, except for the far northern population of Kashmir, an overall lesser incidence of gastric cancer is observed as compared to worldwide incidence. The rates of gastric cancer are higher in the Northern, Northeastern, and certain concentrated regions of the Southern India than the rest of the country. Case-control studies have revealed that high consumption of polished rice and hot and spicy or preserved food, alcohol, and tobacco increase the risk of gastric diseases in South Indian population. Mizoram accounts for the highest rates of gastric cancer after Kashmir [21] in India wherein 30% of cancers in the population are gastric adenocarcinomas. The high risks in this region can be attributed to dietary factors and lifestyle factors with smoking of a local cigarette (Meiziol) and consumption of tobacco smoke infused water (tuibur) being the potent risks in this population [22]. The risk of gastric cancer is higher in less developed countries than in developed countries [20]. This shows a correlation between incidence of gastric cancer and the socio-economic status of the country. High rates of gastric cancer were seen in the Southeast Asian countries like Japan, China, and South Korea [22], the Eastern Europe countries, and certain countries in Latin America [23]. High risk in Southeast Asian countries can be attributed to the high level of carcinogenic nitrates in the preserved food frequently consumed by them [22]. Low risk was seen among populations of North America and Africa. In North America the lifespan probability of developing gastric cancer is 1.5% and mortality risk is 1.0% [11].

## Microsatellite instability (MSI) in gastric cancer

Microsatellites are repetitive DNA sequences which are about 1–6 nucleotides long and are non-randomly spread in both eukaryotic and prokaryotic genomes in the coding and the noncoding regions [24]. Increased rate of mutations like indels in the microsatellite region make it greatly polymorphic in nature. These mutations especially in the coding or regulatory regions change the final phenotype of the organism as a consequence of modification in the expression of associated genes. It is the occurrence of these genomic alterations that is termed as microsatellite instability (MSI). About 15–20% of stomach cancer patients are known to have MSI [25]. For example, upon investigation of Brazilian patients with solitary and sporadic gastric adenocarcinomas, Perez and co-researchers found 21% of them with MSI [26]. National Cancer Institute (NCI) Bethesda has laid out certain guidelines which categorise MSI into three types: high level-MSI (MSI-H), when over 30% of the markers show MSI; low-level MSI (MSI-L), when less than 30% of the markers show MSI; and microsatellite stable (MSS), when MSI is not present at all [27, 28]. Microsatellite markers have been defined and segregated for the purpose of understanding the complex and regulatory regions in the genes.

The MSI-H type of gastric cancer possesses faulty DNA mismatch repair (MMR) process and mutated or methylated MMR genes (hMLH1 and hMSH2) which render them inactive [27]. The MMR system is responsible for rectifying any errors that are made during the replication process in human cells. An alteration in the MMR genes could obstruct the repair of other mutated regions leading to accumulation of mutations and increased susceptibility to carcinogenesis [24]. The occurrence of MSI is owed to the development of errors (like hypermethylation or mutations) in four main MMR genes which include MLH1, MSH2, MSH6, and PMS2 with certain other genes that also play a role less frequently towards MSI [29]. Leite *et al* found 78.7% of MSI gastric cancer samples with hypermethylation of MLH1 promoter [30]. Furthermore, they found that all the samples with this hypermethylation of MLH1 promoter were deficient in the manifestation of PMS2 protein along with that of MLH1.

Not only are the genes of mismatch repair pathway found to be altered, there are numerous other genes belonging to the category of DNA repair/chromatin structure regulation (CHK1, MRE11, RAD50), signal transduction (IGFIIR, TGFβRII), transcriptional regulation (E2F4), miRNA regulation (APAFI), and apoptosis (caspases, BCL10) that are found to be mutated in gastric cancer patients having MSI [25]. For instance, the poly (T) 11 region of MRE11, a protein of the MRN repair complex which is involved in the process of double-strand break (DSB) repair process and in DNA damage response signalling was found to contain mutations in 81% of the total MSI-H gastric cancer cases analysed [31].

In another experimental analysis done by Giovanni Corso and his group, they found 55.6% of MSI GC cases to possess a mutation in at least either one of these genes- EGFR, KRAS, BRAF, PIK3CA, or MLK3- which are members of the mitogen-activated protein kinase (MAPK) cascade and phosphatidylinositol 3-kinase (PI3K) survival pathways [32]. Furthermore, Mario Falchetti *et al* examined the genes regulating cell growth and apoptosis in gastric cancer samples with MSI and found numerous mutations in them with a high incidence of TGFβRII mutations, demonstrating them to be key players in the gastric tumour formation process [33].

One more event linked with tumourigenesis is autophagy which causes type II programmed cell death [34]. Beclin1, an autophagy regulator and a tumour suppressor, is bound by Ultra Violet Radiation Resistance- Associated Gene (UVRAG) to induce autophagy. In addition to these genes, a recent study identified a gene not reported previously—ARID1A, to contain alterations (which include somatic mutations and decreased expression of proteins) in 83% of the analysed MSI GC cases [35]. ARID1A is a chromatin remodelling gene associated with the SWI-SNF complex.

## Epigenetics of gastric cancer

### DNA methylation

DNA methylation refers to addition of a single methyl (-CH3) group to the fifth position of the cytosine ring in the cytosine preceding guanine (CpG])nucleotides usually located in GC rich sites (CpG islands) of the 5’-flanking promoter regions of the genome [36, 37]. The CpG rich regions preceding promoter sequences are normally unmethylated and associated with the active histone mark H3K4me3 which prevents DNA methylation thereby providing access to transcription factors for expression of genes [38]. However, in cancer cells, CpG islands preceding tumour suppressor gene promoters get methylated via inactivation of H3K4me3 and results in the gene being silenced and progression of cancer [39]. Covalent addition of methyl groups at the 5-cytosine position is catalysed by DNA methyltransferases [DNMTs] which use S-adenosyl methionine as the methyl group donor [38, 40]. There are three established DNMTs responsible for DNA methylation: DNMT1, DNMT3a, and DNMT3b. DNMT1 are responsible for maintaining the established correct methylation pattern through cell division in DNA replication [41]. In the DNMT3 family, DNMT3a and DNMT3b are involved in establishing *de novo* methylation patterns during embryogenesis and associate with the replication fork in the late S-phase during replication [41, 42]. Also DNMT3L is responsible for *de novo* methylation but remains catalytically inactive and might cause gene repression even without DNA methylation [43]. DNMTs form a major target area for cancer therapeutics because of their need to be continuously maintained in an active form for the maintenance of the epimutation which can be disrupted by nucleoside/non-nucleoside DNMT inhibitors which prevents DNMT activity by blocking, CYS1226, its catalytic site [44]. DNA methylation can inhibit gene expression by two ways, either by directly interfering with the binding of transcription factors to their specific recognition sites in the promoter region of the genes or indirectly by recruiting proteins that recognise methyl-CpG, called methyl-CpG binding domain proteins (MBDs) which in turn methylate the DNA via histone modification and chromatin remodelling [37, 38]. Both, DNA hypo- and hypermethylation are responsible for cancer development. Global hypomethylation of the genome contributes to oncogene activation and genomic instability which leads to mutagenesis and cancer development. Alternatively local hypermethylation of tumour suppressor genes leads to oncogenesis [41, 42]. DNA methylation, once established, can be inherited by the next generations making it a stable epigenetic mark and hence the most studied epigenetic modification [37, 42, 43].

#### DNA repair genes

The eukaryotic DNA repair machinery is responsible for identifying and repairing any lesion formed in the DNA helix during replication [45]. The repair system includes base excision and nucleotide excision repair pathways, mismatch repair (MMR) pathway, homologous recombination, and genes involved in direct reversal of DNA damage like O-6 methylguanine DNA methyltransferase (MGMT) [39]. Methylation- mediated silencing of the MGMT gene and genes responsible for MMR pathway and homologous recombination have been found to be targets of epigenetic regulation in gastric carcinogenesis [39]. Also a study by Naveed *et al*, identified a transversion in codon 151 (AGC) of the exon 5 segment of MGMT gene which showed negative impact on the interaction between DNA and proteins which may have lead to genomic instability and ultimately cancer [46]. The MGMT gene encodes the O^6^-methylguanine-DNA methyltransferase and plays an important role on DNA repair machinery by removing the cytotoxic O^6^-alkylguanine adducts induced into the DNA by alkylating agents like N-methyl-N9-nitro-N-nitrosoguanidine (MNNG), and N-methyl-N-nitrosourea (MNU) [47]. The mutagenic alkylating agents produce O^6^-methylguanine which mispairs with thymine instead of cytosine during replication. This leads to G: C to A: T mutations where a guanine-cytosine pair gets replaced by an adenine-thymine pair [47]. Hence, promoter hypermethylation mediated inactivation of the MGMT gene causing G to A mutations in the K-ras oncogene has been found to be associated with lymph node metastasis of gastric cancer [37, 48].

The MutL homolog 1 (MLH1) gene in human codes for the DNA MMR protein MLH1 is responsible for mismatch recognition during replication and rectifying the errors in DNA polymerase proofreading activity [39]. Methylation of the promoter region of this gene as well MMR genes coding for MutS homologues 2, 3, and 6 like MSH2, MSH6, or PMS2 shows decrease in the gene activity and is associated mainly with hereditary non-polyposis colon cancer or Lynch syndrome. Some studies on this have also show epigenetic inactivation in gastric cancer [49]. Hypermethylation inactivation of the MLH1 gene also leads to MSI where biallelic inactivation of hMLH1 is required for the expression of the MSI phenotype [39, 49].

#### Tumour suppressor genes and cell cycle regulators

Epigenetic silencing of tumour suppressor genes by promoter CpG island hypermethylation is a common event in all forms of human cancers and hence is a useful epigenetic marker for identification of novel tumour suppressor genes and diagnosis. p16, PRDM5, CHFR, and RASSF1A have been found to be frequently inactivated in gastric carcinogenesis making them useful prognostic markers for the same.

p53 and retinoblastoma (Rb) genes are important tumour suppressor genes and inactivation of which is common in most human cancers. Loss of function of the Rb gene can occur by inactivation of the cyclin dependent kinase inhibitor family of genes, the INK4 family, in which p16^INK4a^ inactivation is closely associated with gastric carcinogenesis [50]. The p16INK4a gene is responsible for inhibiting the association between Cdk 4/6 and cyclin D thereby blocking the Cdk4/6 mediated phosphorylation of Rb protein and consequently preventing the G1-to-S transition in cells [51]. Loss of p16 activity by point mutations and homozygous deletions is common in most types of cancer, however, further studies suggest *de novo* methylation of the 3’ end promoter region of specific CpG sites surrounding the ATG initiation codon of p16 and hypermethylation of exon 1α coding region as predominant mechanisms of p16 inactivation during tumourigenesis of gastric carcinoma [51]. The 3’promoter hypermethylation is conserved to specific CpG sites associated with putative transcriptional start sites and is a late event in progression of gastric carcinoma as some p16-negative tumour areas were found to co-exist with p16-positive tumour regions in a study by Song *et al* [51]. Aberrant methylation of p16 has been associated with MSI where MSI positive tumours were found in gastric cancer cell lines expressing CpG island methylator phenotype [52].

A portion of p16^INK4a^ encodes for another tumour suppressor gene p14^ARF^ in humans which prevents MDM2 mediated degradation of p53 and acts as a cell cycle regulator by inducing G_1_ to G_2_ phase arrest thereby resulting in tumour prevention [50]. The p14^ARF^ gene activity is independent of the activity of p16^INK4a^ with the gene expression being controlled by exon 1β promoter region. p14^ARF^ promoter hypermethylation is not dependant on p16^INK4a^ methylation status, however, p14^ARF^ and p53 inactivation can be found simultaneously in the same tumour [50].

PR domain zinc finger protein 5 (PRDM5), a member of the Kruppel-like zinc finger family is an important tumour suppressor gene and has been found to be methylated in gastric human carcinomas like [37]. It exerts its tumour preventing properties by inhibiting the WNT/β-catenin signalling pathway thereby causing G2/M cell cycle arrest and inducing apoptosis in tumour cells [37, 53]. The inhibition of the Wnt/β-catenin pathway by PRDM5 is mainly through upregulation of DKK1, DKK2, and WNT5A and downregulation of the CCND1 promoter activity, a downstream target oncogene of WNT/β-catenin signalling, and a mitogenic signal sensor of the same [53]. Certain studies also reveal that PRDM5 expression downregulate several oncogenes like CDK4, TWIST1, and MDM2 thereby preventing tumour progression [53].

#### Apoptotic genes

The advent of chemotherapy has helped in prolonging the survival time for patients with metastatic and recurring cancer, however, gastric cancer still rates as the second most common cause of cancer related deaths in the world owing to its insufficient effectiveness to chemotherapy and absence of reliable markers to predict the response of chemotherapy on gastric cancer [54]. Chemotherapy generally induces apoptosis and damage to the DNA in the progressing tumours, i.e., inactivation of the apoptotic genes like Bcl-2/adenovirus E1B 19 kDa-interacting protein 3 (BNIP3) and death-associated protein kinase (DAPK) by methylation generates chemo-resistance in gastric cancer patients [54].

BNIP3 is a pro-apoptotic member of the Bcl-2 family induced by hypoxia in anoxic regions of tumours and is antagonistic to pro-survival proteins like Bcl-2 and Bcl-xl. DAPK is a Ca^2+^/calmodulin-dependant enzyme with serine/threonine activity and functions as an apoptotic regulator as a part of the p53-dependant apoptotic pathway that operates via p19^ARF^ [54]. Inactivation of these genes by promoter hypermethylation is a common event in gastric carcinogenesis which makes them important prognostic markers of chemoresistance in gastric cancer.

#### Transcriptional regulators

RUNX3 is a mammalian runt-related transcription factor which has been identified as a tumour suppressor in the recent studies [55]. It is an important growth regulator in gastric epithelia and an important target of TGF-β signalling that plays a major role in mammalian development [56]. Inactivation of this gene in gastric cancer is mainly because of hypermethylation of the promoter region and hemizygous deletion [55]. RUNX3 methylation is mostly cancer specific and more frequent in the non-neoplastic gastric epithelia of patients above 77 years of age. This shows that RUNX3 methylation is age related and its silencing is associated with advanced stage of gastric cancer. It is also related to stage of tumour invasion and distant metastasis making it an important prognostic marker [55].

CDH5 is a non-histone chromosomal protein belonging to the SWI2/SNF2-related superfamily of ATPases and a potent tumour suppressor gene [57]. It stabilises the p53 apoptotic pathway in almost 50% of human cancers by promoting transcription of p16^ARF^ and p19^INK4A^ genes [58]. Hence downregulation of this gene by promoter hypermethylation may compromise the p53 signalling and lead to development of cancer [57].

#### Signalling pathway regulators

RASSF1A is an important tumour suppressor gene which plays a role in the microtubule and genomic stability by regulating the Ras signalling pathway [59]. Hypermethylation in the CpG island of the RASSF1A promoter causing loss or reduced gene expression is correlated to gastric tumourigenesis which makes it a potential molecular marker for detection and prognosis of gastric adenocarcinomas. Furthermore, its epigenetic inactivation is also associated with TNM stage and poor prognosis in patients with gastric cancer [59].

Abnormal activation of the Wnt/β-catenin signalling is frequently found in gastric cancer as quite a few target genes of this signalling pathway are associated with tumourigenesis. Dkk-3 gene acts as an inhibitor of Wnt signalling by targeting β-catenin for phosphorylation and degradation by ubiquitination. Thus, inactivation of this gene by promoter hypermethylation contributes to activation of Wnt signalling and promotion of tumour by aberrant cell proliferation and differentiation [60]. In a study by Yu *et al* [60] out of 17 gastric cancer cell lines studied, Dkk-3 inactivation was found in 12 (70.6%) of them and this inactivation was correlated to promoter methylation that could be restored by treatment with demethylation agent like 5-aza-2’-deoxycytidine in combination with trichostatin A. The study also demonstrated methylation of Dkk-3 as an independent prediction marker in gastric cancer and related to poor disease survival in most aggressive type gastric cancer [60].

Another important tumour suppressor gene, ADAMTS9, was found to be frequently silenced in gastric cancer samples thereby making it an important prognostic marker. It is a mammalian metalloprotease enzyme with proteolytic activity and is a member of the ADAMTS protease family that is localised to the extracellular matrix [61]. It inhibits tumour growth by blocking the oncogenic AKT/mTOR signalling pathway and downregulating the cell proliferation and angiogenesis and upregulating cell apoptosis [61]. MSP and BGS assays showed promoter methylation of ADAMTS9 in 29.2% gastric cancer samples in a study by Du *et al* [61]. The study also revealed that this methylation is associated with shortened survival in gastric cancer patients.

### Histone modifications

Epigenetic changes are also associated with chromatin remodelling of which histone modifications form an important part. The eukaryotic chromosome is complex and consists of nearly 146 base pairs of DNA wrapped around a histone octamer consisting of two of each H2A, H2B, H3, and H4 histone proteins called a nucleosome [62]. Histones are highly alkaline and enriched with lysine and arginine residues which impart positive charge to them and hence can readily bind to negatively charged DNA. Thus it can be predicted that histone modification could affect the interaction between DNA and histones thereby regulating gene expression and tumour development [63]. Modification of the N-terminus of the histone core proteins (H2A, H2B, H3 and H4) by multiple covalent post-translational modifications like acetylation, methylation, and phosphorylation forms the basis of histone modification [63]. Recent studies on histone modifications with more focus on acetylation, methylation, and phosphorylation show that acetylation and methylation of H3 or H4 are more common events [64].

#### Histone acetylation

The configuration of nucleosomes is altered by acetylation or deacetylation of the N-terminal group of lysine residues on histones which directly influences the transcriptional status of the genes [62]. Acetylation of the lysines by histone acetylase (HATs) causes transfer of acetyl group from acetyl Co-A to the specific lysine residue on the terminal end of the core protein [63]. This result in an open chromatin structure and activated gene transcription by removal of the positive charge on histones which facilitated binding to negatively charged DNA and maintenance of the transcription repression. Histone deacetylases (HDAC) on the other hand antagonises this mechanism and reverses this process by removing the acetyl group from lysine and silencing the gene expression. Hence irregular acetylation or deacetylation may result in neoplastic transformation by activation of oncogenes or inactivation of tumour suppressor genes [64].

Histone acetylation has also been found to play a major role in gastric carcinogenesis. H3 acetylation on the promoter region silencing tumour suppressor gene P21 WAP1/CIP1, and downregulation of p16, CDH1, MLH1 by histone modification prove that there exists interplay between histone and DNA chemical modifications in development of gastric cancer [63, 65]

#### Histone methylation

Histone methyltransferase (HMT) which includes histone arginine methyltransferase (HRMT) and histone lysine methyltransferase (HKMT) are responsible for methylation of specific sites in lysine or arginine of H3 or H4 [63]. Similar to acetylation histone methylation is also a reversible process with its antagonist being histone demethylase (HDM). However, unlike acetylation, histone methylation is variant with different sites and types having different effect on gene expression. On histone 3 Lys-4, methylation results in open chromatin structure and activation of transcription whereas Lys-9 methylation represses transcription because of the condensed form of chromatin [64].

There is interplay between histone modifications and other epigenetic changes that together contribute to tumourigeneis. In gastric cancer apart from the broadly studied DNA methylation of the promoter region of p16, acetylation and dimethylation of Lys-9 residues on H3 also contributes to silencing of the tumour suppressor gene [63].

As seen earlier in the article, RUNX3 is an important transcriptional regulator which found to be frequently hypermethylated at the promoter regions in gastric cancer. In addition to that, hypoxia induced Lys-9 methylation, Lys-4 demethylation, and deacetylation of H3 are early events in RUNX3 downregulation which facilitates gene silencing by cytosine methylation [65]. Thus studies reveal that promoter hypermethylation is not just the only mechanism of inactivation if this TSG and that histone deacetylation and methylation also play an important role in the silencing of this gene.

### RNA in gastric cancer

miRNA, consisting of nearly 22 nucleotides, forms miRNA-induced silencing complex in order to degrade specific mRNA molecules or obstruct their translation into functional proteins [66]. On the other hand, lncRNA have more than 200 nucleotides and is a major part of the human transcriptome.

There is evidence that miRNA dysregulation is caused by methylation of its promoter sites. For instance, methylation in its promoter reduced the expression of miR-137 (a tumour suppressor) in gastric cancer. Similarly, miR-335, miR-495, miR-9, miR-10b, miR-219-2-3p, miR-212, miR-941, and miR-1247 were found to be silenced because of methylation [66]. Studies have also shown that in gastric cancers, these miRNAs may act as both oncogenes as in the case of miR-19a and as tumour suppressor genes like miR-874 [67]. Junming *et al* found miR-20b, miR-20a, miR-17, miR-106a, miR-18a, miR-21, miR-106b, miR-18b, miR-421, miR-340, miR-19a, and miR-368 to be highly expressed in GC tissues [68].

While a lot is known about the underlying mechanisms of miRNA dysregulation, the possible reason for the abnormal function of lncRNAs is yet to be understood [66]. In recent times, these lncRNAs have become known to have regulatory effects in several essential biological processes which might also have some role to play in the process of tumourigenesis. Their faulty expression is also found to cause GC, for example, according to the work done by Song and his team, H19 has high expression in gastric cancer tissues than in normal ones. They also show that instead of being overexpressed in hepatocarcinoma and prostate cancer, H19 was present in lower levels suggestive of the fact that it may act as both an oncogene and a tumour suppressor gene [66]. In another work by Peng *et al*, Maternally Expressed Gene 3 (MEG3) is found in lower levels in GC samples with a link to metastatic GC. They also showed that MEG3 had inhibitory effects on cell proliferation, migration, and invasion and had a positive effect on apoptosis [69].

Even though all the phases of cancer are affected by the non-coding RNAs with them being either a tumour promoter or a repressor, the underlying pathway leading to their unusual expression is not yet known [66]. Hence, an in depth study into this area needs to be done as these could act as suitable markers in detecting GCs.

## Conclusion

Gastric cancer is a disease with a high mortality rate with nearly three quarter of a million deaths annually. However, a significant decline has been seen over the last decade with the advancement of chemotherapy regimens, incorporation of low toxicity oral drugs, and early diagnostic tools. While *H. pylori* infection is considered to be one of the significant causative factors for gastric cancer, there are other important aspects to the disease, with epigenetic variation being a significant contributor. Methylation of genes forms the basis of most of these epigenetic variations and also of other parameters like MSI which is common in gastric cancer patients. Epigenetics including methylation of DNA repair genes serve as prognostic markers in human gastric cancer. However, apoptotic genes like BNIP3 and DNA repair gene MLH1 are chemo-sensitive prognostic markers. Also, the promoter region hypermethylation of Dkk-3, CDH-5, DDAPK, p16, and RASSF1A genes are individual biomarkers useful in predicting the clinical features of gastric cancer patients. Apart from DNA methylation, histone modifications, and non- coding RNA have also been found to play an important role in cancer progression because of which detailed research is needed to be done for understanding their role as prognostic and diagnostic markers. Inhibitors of HDAC which can increase acetylation level and maintain the tumour suppressor genes in an activated state are promising therapeutic compounds [64]. The non-coding RNAs are involved in numerous pathways associated with cancer development, either as suppressors or promoters, though knowledge into the exact mechanism involved remains elusive in a lot of cases.

The present review reveals an important link between the environmental factors, epigenetic variations, and microsatellite instability(MSI) in gastric carcinogenesis. An in depth study of these epigenetic alterations in gastric cancers may be a great aid to develop novel therapeutic markers and develop better diagnostic tools. Also, understanding epigenetic mechanisms holds great promise for cancer prevention, detection, and therapy.

## Disclosure of potential conflicts of interest

Not applicable.

## Research involving human participants and/or animals

**Informed consent**

Not applicable.
